# Supplementary figures and images for: COWAVE: A labelled COVID-19 wave dataset for building predictive models
Source: PLoS One. 2023 Jul 25;18(7):e0284076. doi: 10.1371/journal.pone.0284076 (PMC10368260; doi:10.1371/journal.pone.0284076)

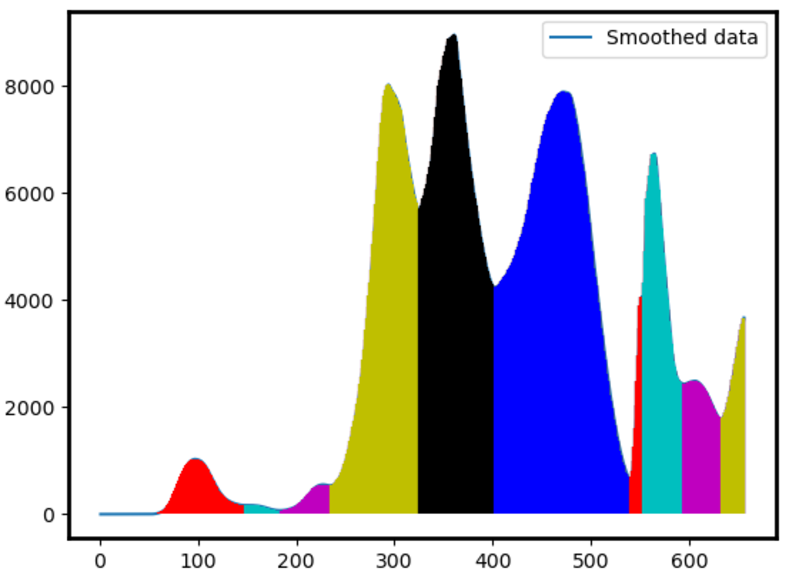

Supplement: S1 Fig — The Y-axis shows the number of cases, for a given date. The X-axis ranges from 3-Jan-2020 (Day 0) to 20-Oct-2021 (Day 655). Here, regions of different colours indicate different unique waves. (TIF) [file pone.0284076.s001.tif]

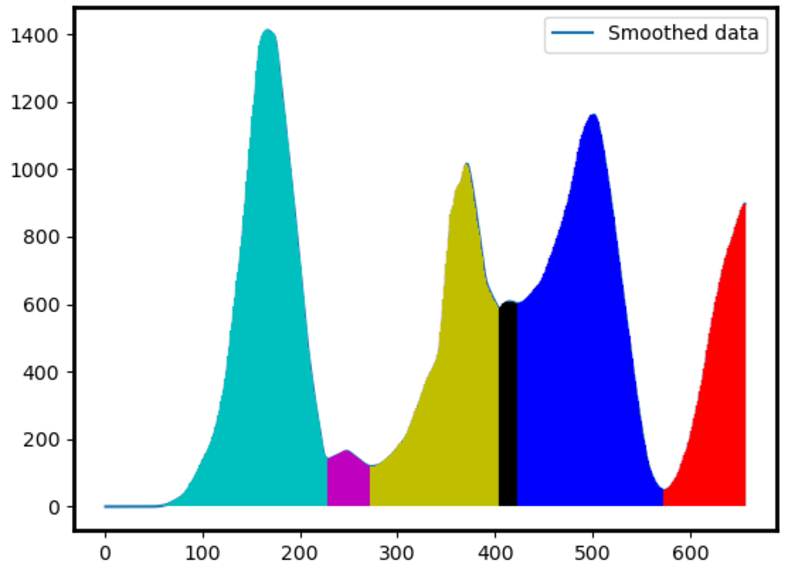

Supplement: S2 Fig — The Y-axis shows the number of cases, for a given date. The X-axis ranges from 3-Jan-2020 (Day 0) to 20-Oct-2021 (Day 655). Here, regions of different colours indicate different unique waves. (TIF) [file pone.0284076.s002.tif]

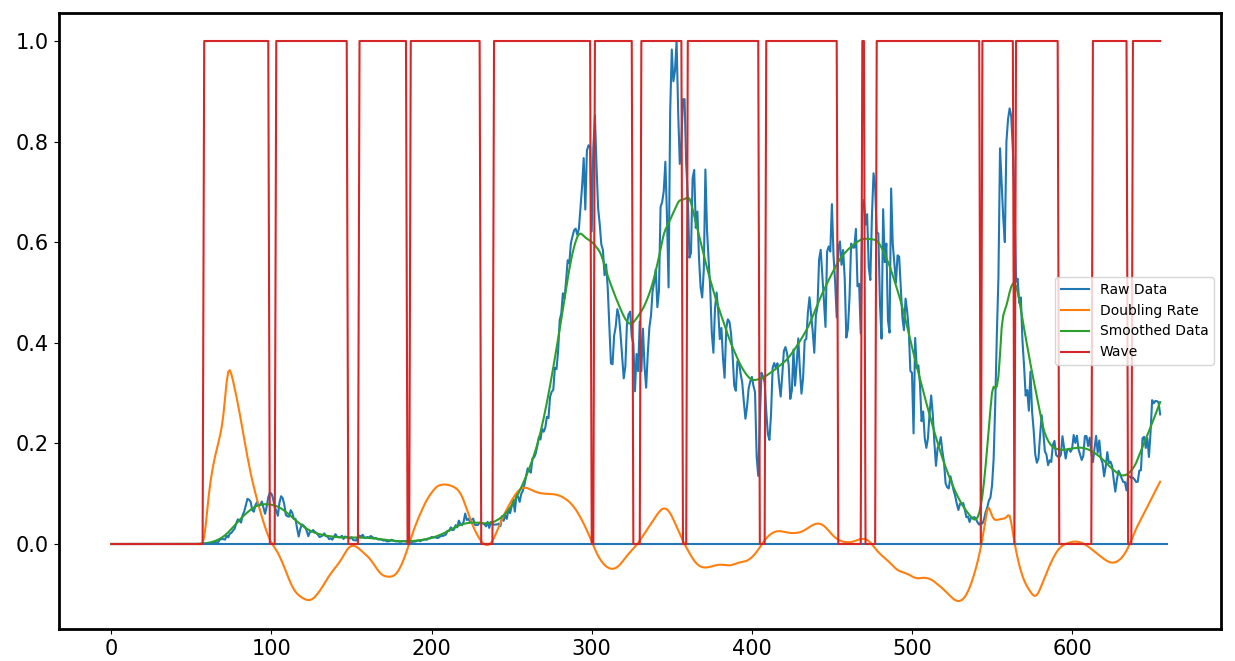

Supplement: S3 Fig — The Y-axis shows the number of cases, for a given date. The X-axis ranges from 3-Jan-2020 (Day 0) to 20-Oct-2021 (Day 655). The blue curve represents the raw data; the green curve, smoothed data; the orange curve, the LOWESS smoothed doubling rate, and the red curve indicates whether the day is part of a wave. (TIF) [file pone.0284076.s003.tif]

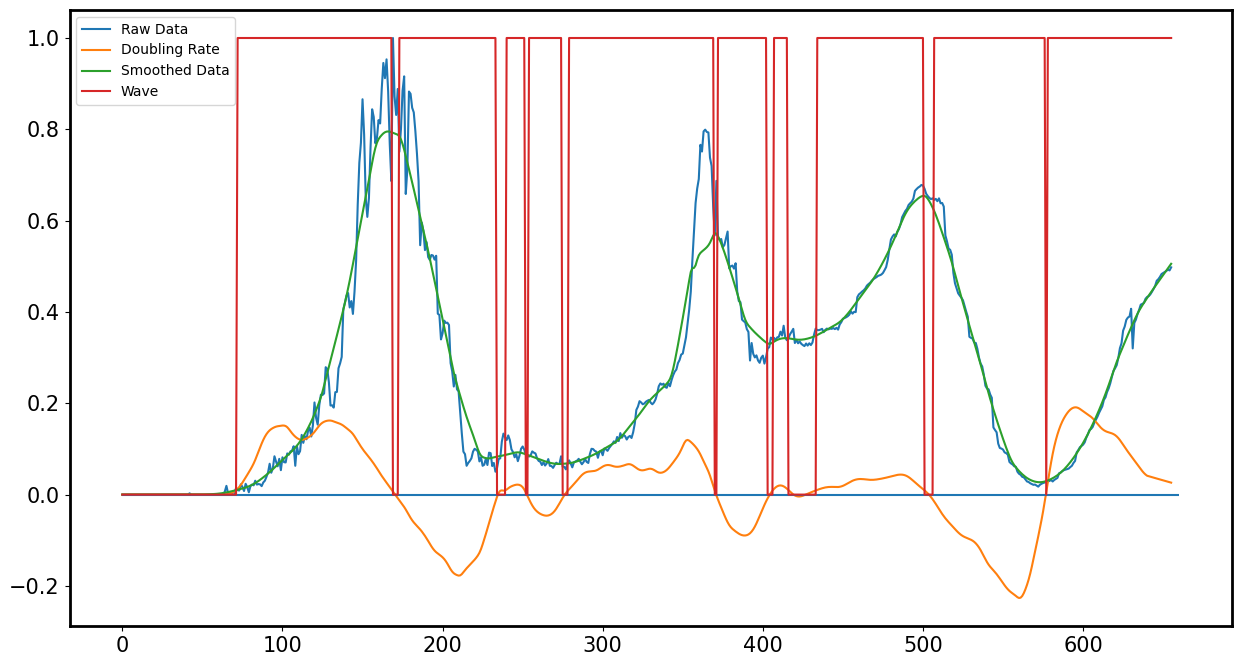

Supplement: S4 Fig — The Y-axis shows the number of cases, for a given date. The X-axis ranges from 3-Jan-2020 (Day 0) to 20-Oct-2021 (Day 655). The blue curve represents the raw data; the green curve, smoothed data; the orange curve, the LOWESS smoothed doubling rate, and the red curve indicates whether the day is part of a wave. (TIF) [file pone.0284076.s004.tif]

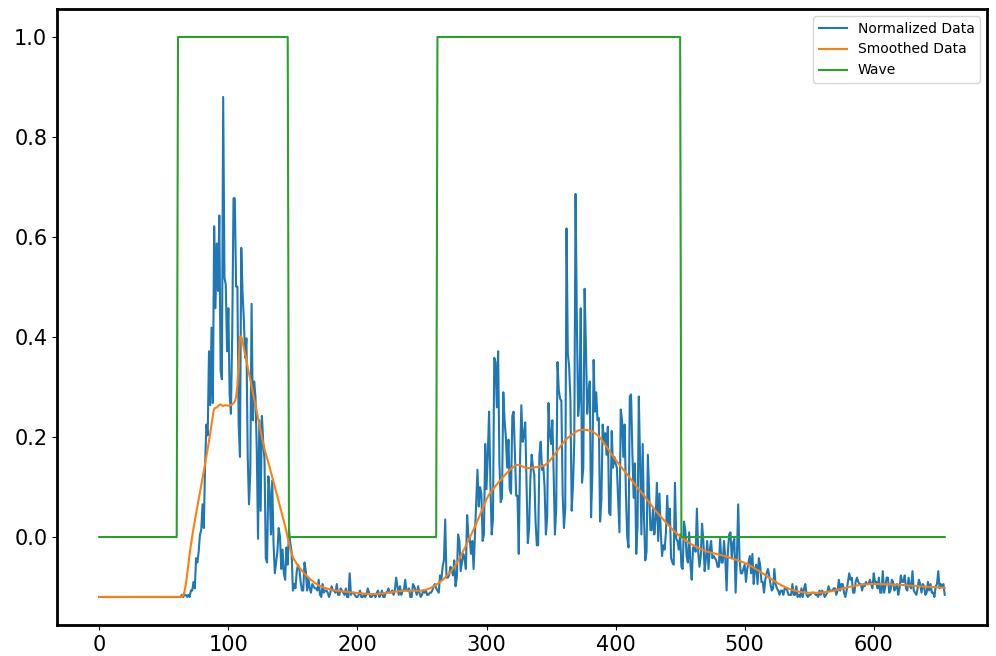

Supplement: S5 Fig — The Y-axis shows the number of cases, for a given date. The X-axis ranges from 3-Jan-2020 (Day 0) to 20-Oct-2021 (Day 655). The blue curve represents the raw data; the orange curve, smoothed data, and the green curve indicates whether the day is part of a wave. (TIF) [file pone.0284076.s005.tif]

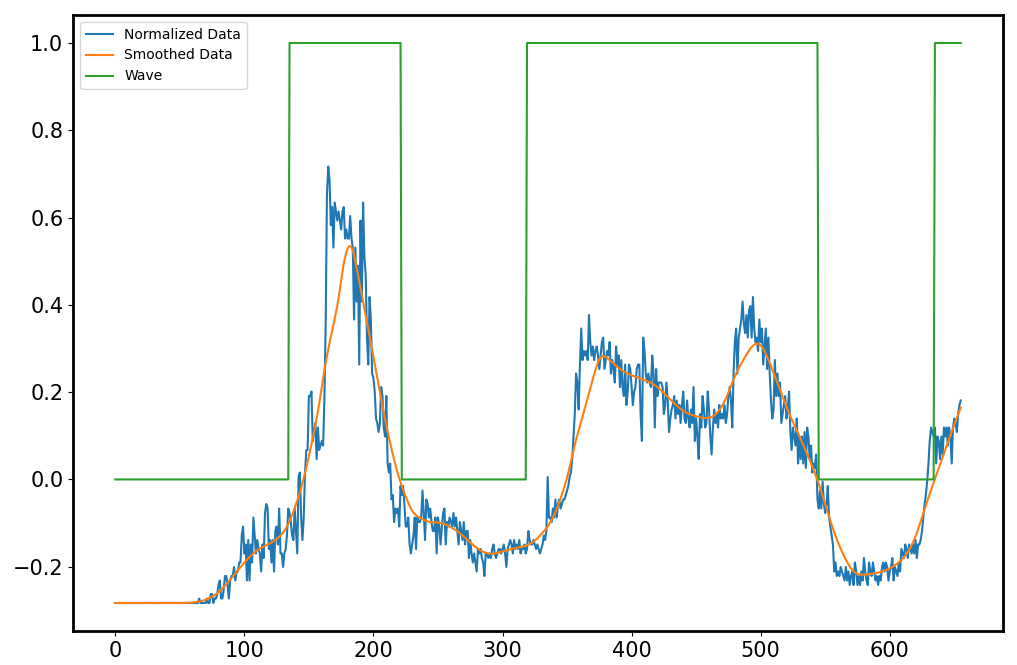

Supplement: S6 Fig — The Y-axis shows the number of cases, for a given date. The X-axis ranges from 3-Jan-2020 (Day 0) to 20-Oct-2021 (Day 655). The blue curve represents the raw data; the orange curve, smoothed data, and the green curve indicates whether the day is part of a wave. (TIF) [file pone.0284076.s006.tif]
